# Use of Subdural Evacuating Port System Following Open Craniotomy with Excision of Native Dura and Membranes for Management of Chronic Subdural Hematoma

**DOI:** 10.7759/cureus.1197

**Published:** 2017-04-26

**Authors:** Tene Cage, Ashley Bach, Michael W. McDermott

**Affiliations:** 1 Department of Neurological Surgery, University of California, San Francisco; 2 School of Medicine, University of California, San Francisco

**Keywords:** subdural hematoma, craniotomy, subdural drain

## Abstract

An 86-year-old woman was admitted to the intensive care unit with a chronic subdural hematoma (CSDH) and rapid onset of worsening neurological symptoms. She was taken to the operating room for a mini-craniotomy for evacuation of the CSDH including excision of the dura and CSDH membrane. Postoperatively, a subdural evacuation port system (SEPS) was integrated into the craniotomy site and left in place rather than a traditional subdural catheter drain to evacuate the subdural space postoperatively. The patient had a good recovery and improvement of symptoms after evacuation and remained clinically well after the SEPS was removed. We offer the technique of dura and CSDH membrane excision plus SEPS drain as an effective postoperative alternative to the standard craniotomy leaving the native dura intact with traditional subdural drain that overlies the cortical surface of the brain in treating patients with CSDH.

## Introduction

The incidence of chronic subdural hematomas (CSDH) in the general population has been rising, which is in part due to an increase in the elderly population and an increase in the use of oral antiplatelet and anticoagulation agents in this group of patients [1-2]. The incidence of CSDH has been reported to be approximately 14 per 100,000 person years [1,3]. This is almost double the incidence of the most common primary brain tumor in adults, meningioma, which is 7.8 per 100,000 person years. Patients often live with chronic, subacute, or mixed subacute on chronic SDHs in the community and do not present to medical attention until they become symptomatic. Symptoms can mimic any neurological condition and often include headaches, somnolence, contralateral body weakness, or language difficulties, if the SDH is causing mass effect on the dominant language hemisphere and can include non-localizing symptoms resembling dementia. For the symptomatic patient, neurosurgical intervention for chronic and subacute on CSDHs include bedside subdural evacuating port system (SEPS), operative burr hole(s), or craniotomies to remove the blood products. Following evacuation of the SDH via either burr holes or craniotomy, the literature supports leaving a subdural drain in place postoperatively to avoid re-accumulation of SDH [4-5]. The type of drain varies among neurosurgeons, but the practice of leaving a drain postoperatively is supported. A subdural drain usually means that a rubber drain is left in the subdural space overlying the brain. In a patient where the brain is atrophic due to age or otherwise, inserting a drain in this space may cause increased bleeding due to tearing of a bridging vein or a friable cortical vessel. The SEPS system, in contrast, allows for continued subdural drainage without the physical drain tubing lying over the brain surface. In addition, when performing a craniotomy for a recurrent CSDH or subacute on CSDH, the practice of peeling away the outer organized membrane of the hematoma that is attached to the dura can lead to recurrent bleeding and acute deterioration. Rather than performing this “stripping” of the outer membrane, another option is to excise the outer membrane with the dura and reconstruct the dura with a dural substitute xenograft.

Here, we present the case of a patient who required a craniotomy for subacute on CSDH evacuation, excision of the dura and outer CSDH membrane, and placement of SEPS drain integrated into the craniotomy for postoperative subdural drainage. The patient had a good clinical result without reaccumulation of the CSDH. This report documents the procedure of membrane and dura excision with placement of a SEPS drain as a post-craniotomy drainage system for CSDH management.

In accordance with the University of California San Francisco Institutional Review Board (IRB), IRB approval is waived because this is a technical report describing a case study of one patient. There is no identifying patient information included in this study and therefore no patient consent was required.

## Technical report

### Presentation

**Figure 1:**
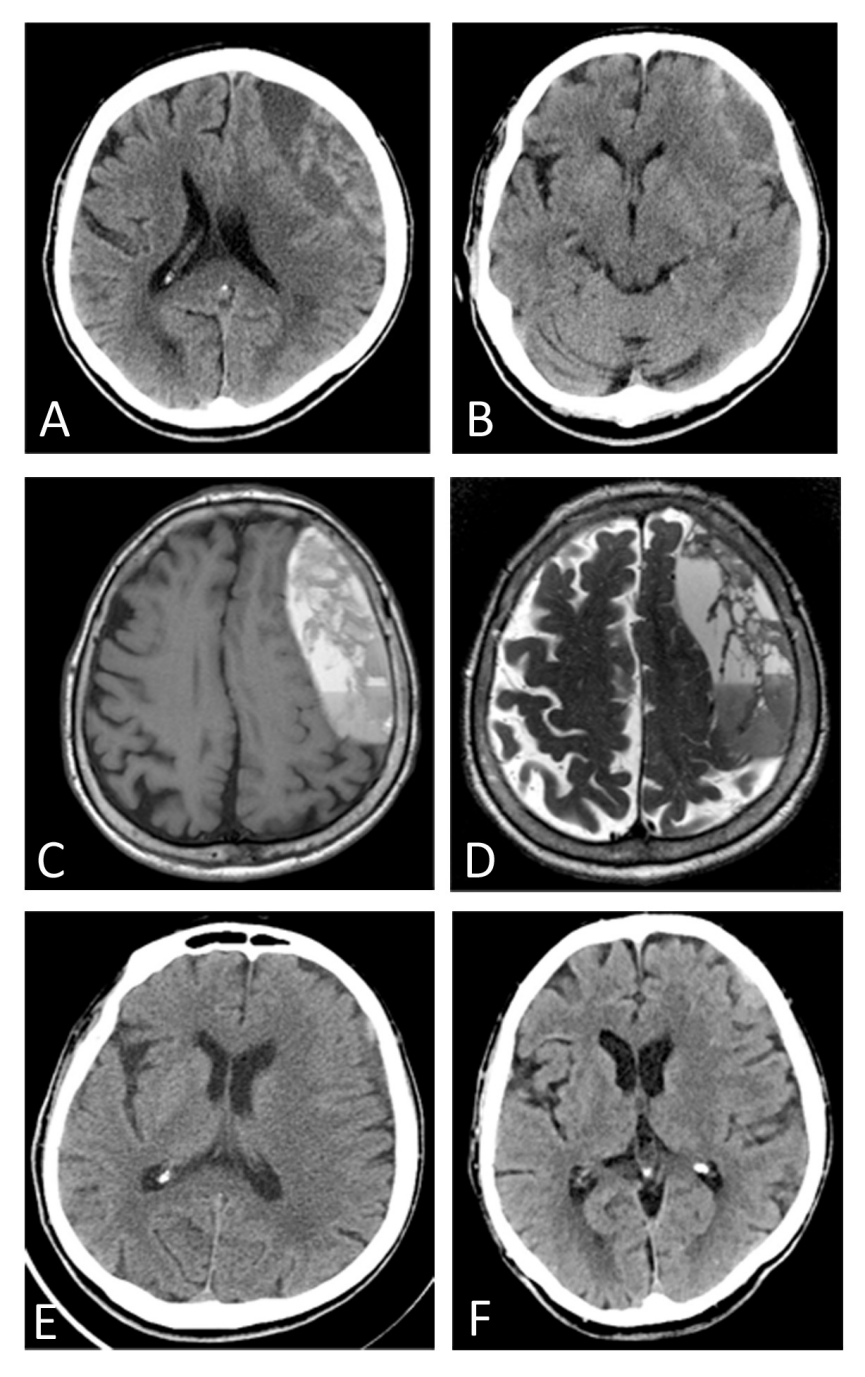
Pre and postoperative imaging of chronic subdural hematoma Preoperative non-contrast CT scan (A,B), T1 weighted (C), and T2-weighted (D) MRI scans demonstrating subacute on early chronic left convexity subdural hematoma causing local mass effect and left to right midline shift. Postoperative non-contrast CT scans at one month (E) and two months (F) postoperative showing signficant improvement in evacuation of hematoma and resolution of midline shift and local mass effect.

An 86-year-old woman was brought to the emergency department for evaluation of worsening word finding difficulty and generalized weakness. The patient’s son stated that she had suffered two falls recently, the first was approximately two months prior to presentation and the second was four weeks prior to presentation. The patient’s past medical history included diabetes, hyperlipidemia, and a remote lumbar fusion for back pain. She had taken aspirin the day prior to presentation and she was not on any other anticoagulation or antiplatelet medications. Non-contrast computed tomography (CT) scan of the head showed a 3 cm left frontal mixed density extra-axial fluid collection concerning for subacute on CSDH causing local mass effect as well as 3.5 mm of left to right midline shift (Figures 1A-1B).

### Examination

On examination at the initial time of evaluation, the patient was awake and attentive, but was oriented only to person and her speech output was markedly slowed. Though she was able to name objects, count, and calculate, she displayed some difficulty with repetition. Her cranial nerve exam was intact but motor exam revealed weakness in the right upper and lower extremities. She had no signs of external trauma and denied any localized pain.

### Procedures

The patient was admitted to the neurosurgical intensive care unit (ICU) for close monitoring for neurological status changes. Since she had taken aspirin the day prior to admission, she was given one unit of platelets. Due to the subacute and chronic appearance of the extra-axial collection, no antiepileptic medications were initiated at this time. Because of the complex septated and loculated appearance of the extra-axial collection on CT scan, she underwent a magnetic resonance imaging (MRI) of the brain on hospital day #1 to further evaluate the collection. The MRI confirmed subacute on CSDH (Figures 1C-1D). Overnight on hospital day #1, her neurologic exam began to decline and was marked by somnolence and significantly decreased verbal output. Repeat head CT demonstrated the stable large subacute on CSDH with no evidence of acute blood. Therefore, she was taken to the operating room for a mini-craniotomy to evacuate the SDH.

**Figure 2:**
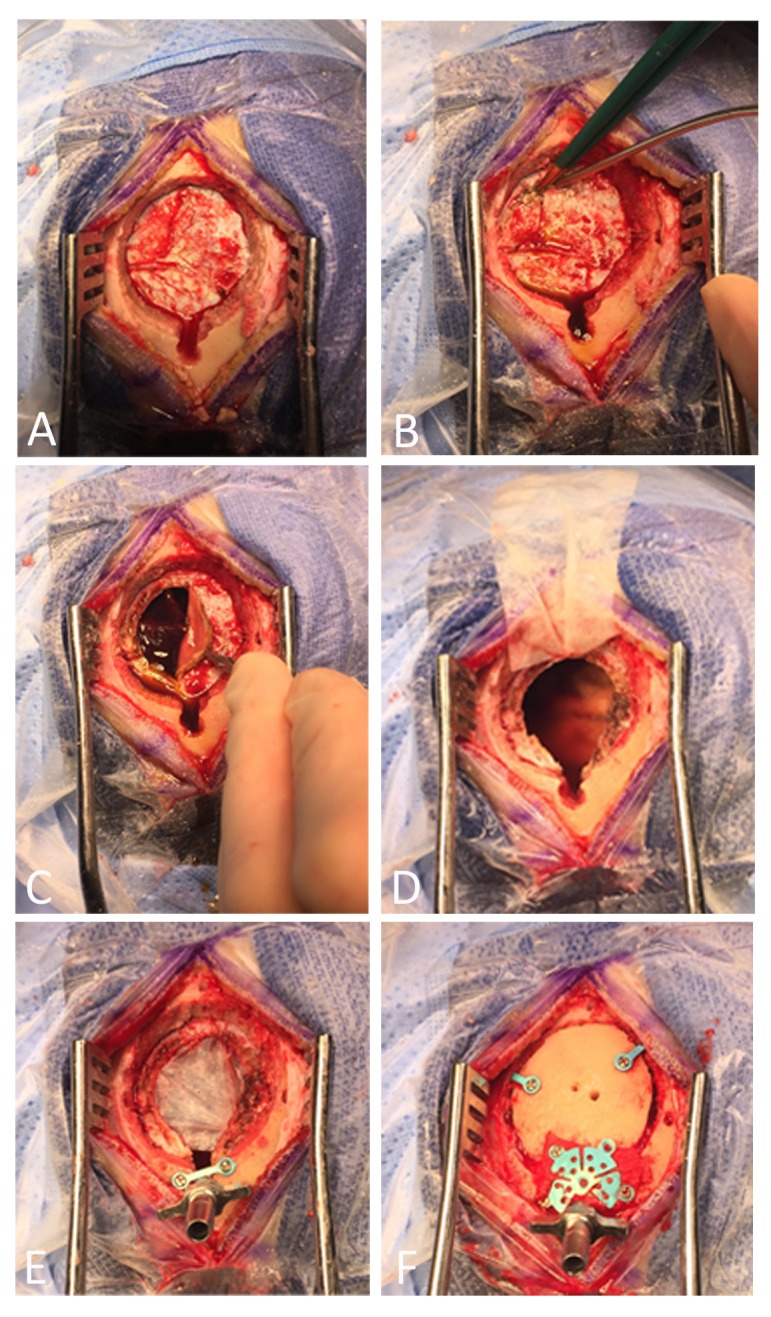
Operative technique Operative technique of mini-craniotomy for CSDH evacuation with SEPS drain as postoperative drainage system. (A) The SEPS twist-drill hole is drilled first, then a mini-craniotomy flap is elevated adjacent to the twist-drill site. The two are connected removing bone with a Kerrison rongeur. (B) The dura under the craniotomy is coagulated then excised (C) to reveal underlying CSDH membranes and fluid. (D) Once the dural flap is excised and the CSDH is evacuated with copious irrigation, a dural substitute is cut to size and sewn in place over the defect left from the excised dura. (E) The SEPS bolt is inserted and a two-hole cranial plate can be used to help hold the bolt in place if needed. (F) The craniotomy flap is replaced and held in place with two two-hole plates and a burr hole cover with one flange removed to sit around the SEPS bolt.

The patient was positioned supine and her head was placed in a Mayfield pin head holding device (Integra, NJ, USA). After the hair was shaved and the skin prepped and draped in sterile fashion, a linear incision was made over the left frontal region overlying the subdural fluid collection. A cerebellar retractor was placed to expose the underlying skull. Once the skull was exposed, the hand drill from the SEPS system kit was used to drill a hole for the SEPS to remain as the subdural drain postoperatively. Once that hole was drilled, a burr hole was drilled close to but not in continuity with the SEPS hole. Then a craniotome was used to turn a mini-craniotomy flap adjacent to the SEPS twist-drill site. The craniotomy flap was elevated and the small island of bone between the mini-craniotomy and the twist-drill site was removed using a Kerrison rongeur (Kerrison King, TX, USA). Care was taken to leave approximately 270 degrees of the SEPS twist drill hole intact and open the remaining 90 degrees to the mini-craniotomy (Figure 2A).

With the dura overlying the subdural hematoma exposed, a circular patch of dura and the organized outer CSDH membrane was then excised to reveal underlying chronic subdural hematoma and subdural membranes (Figures 2B-2C). Then, the opening in the dura was continued using the bipolar to coagulate both the dura and outer membrane at the same time before cutting the dura with scissors. The excision of the dura was made to be continuous with the area of the twist-drill site to ensure that the SEPS drain would drain the subdural space. Next, the subdural hematoma was evacuated under direct visualization and the subdural space was copiously irrigated with Lactated Ringers solution. Then, a patch of xenograft dural substitute was cut to size and sewn into the dural defect to replace the large piece of native dura that was removed leaving an opening in the dura under the twist-drill site (Figure 2D).

Next, the SEPS metal bolt was screwed into place into the twist drill site. The bolt should sit snugly in the hole, but if the opening between the craniotomy and the twist drill site was enlarged too much with the Kerrison rongeur, a small two-hole cranial plate can be used to span the open side of the twist drill site for additional support to hold the bolt in place (Figure 2E). The bone flap was then plated using two small two-hole plates and a small burr hole cover over the burr hole site with one flange removed to accommodate the SEPS bolt. Finally, a small piece of GelFoam (Pfizer, NY, USA) was placed under the burr hole site and the mini-craniotomy bone flap was replaced and screwed into place (Figure 2F). The galea was then re-approximated with vicryl sutures and the skin closed with a running non-absorbable monofilament suture. The rubber tubing was connected to the SEPS bolt and the bulb was placed to suction on the distal end of the tubing. This creates a postoperative subdural drainage system that obviates the need for a rubber drain directly in the subdural space on the brain which may cause additional bleeding.

### Outcome

Postoperatively, the patient recovered in the ICU and she improved clinically to return to her baseline neurologic status. The SEPS drain was removed on postoperative day 2 when the subdural collection was resolved and midline shift improved. She was discharged to home in good condition. At one month and two month follow-ups the patient remained asymptomatic at her neurologic baseline and a CT scan confirmed resolution of the midline shift and significant decrease in size of the left frontal chronic subdural fluid collection (Figures 1E-1F).

## Discussion

This patient’s case demonstrates a two-step alternative option for treatment of CSDH: (1) excision of both the dura and outer CSDH membrane as a single layer in combination with (2) use of a SEPS drain integrated into the craniotomy for postoperative drainage of the subdural space following surgical evacuation of a symptomatic CSDH. The excision of the outer membrane with the dura using coagulation of both tissues together may reduce the chance of acute postoperative bleeding as compared to opening of the dura, “stripping” the outer membrane off of the overlying dura, and primary closure of the native dura. Typically, the SEPS drain is used as a stand-alone device placed at the bedside to drain a liquefied subdural fluid collection. Here, the authors use the device as a postoperative drainage system as an adjunct to a burr hole or mini-craniotomy.

The literature supports leaving a subdural drain in place after evacuation of CSDH to help avoid re-accumulation of fluid [4-5]. However, there is concern that leaving a rubber Jackson-Pratt drain or other catheter in the subdural space may cause a bridging vein or cortical vessel to be torn either during placement or removal of the drain, which may thereby cause an iatrogenic acute subdural hematoma. Alternatively, a short rubber drain that does not contact the cortical surface but instead extends only a few centimeters into the subdural space is sometimes left in the burr hole for continued postoperative drainage. This technique carries the risk of the tubing being unintentionally advanced deeper into the subdural space during the subsequent steps of the operation which again, brings risk of injury to the cortical surface and associated bleeding. The SEPS device provides postoperative drainage of the subdural space without a rubber drain or catheter lying over the brain or in the subdural space that could potentially cause bleeding from shearing of subdural and cortical vessels.

Though our patient was able to be safely taken to the operating room when her symptoms worsened due to the mass effect of the CSDH on the brain, other patients may present with mild, stable neurologic deficits and cranial imaging confirming CSDH. For these patients, it is reasonable to place a SEPS at the bedside as the initial intervention. If the SEPS is unsuccessful in evacuating the subdural fluid, the patient should then be taken to the operating room for burr holes or craniotomy and the existing SEPS twist-drill site can be carefully incorporated into the burr hole or craniotomy. In this case, at the conclusion of the operation, a new SEPS bolt and drain should be used as the postoperative subdural space drain. Prior to removal of the SEPS drain postoperatively, the output should be carefully monitored and a CT scan obtained to evaluate the remaining subdural collection to determine if further intervention such as repeat burr holes or craniotomy is necessary for CSDH resolution.

## Conclusions

CSDH is a growing problem in the neurosurgical patient population. It is well established that the fluid must be evacuated and, following an open burr hole for evacuation, a drain should be left postoperatively to avoid re-accumulation of fluid. If there is a subacute on CSDH, or recurrence for a second time of a CSDH, then craniotomy may be indicated. When using a craniotomy, excision of the dura and the outer membrane after coagulation of both tissues followed by hematoma evacuation and then suturing in of a dural substitute may reduce the chance of postoperative bleeding. However, the technique described here does have some limitations. Though the dural excistion, placement of a dural substitute, and SEPS insertion are not technically challenging to perform, together these steps may lengthen the operative time associated with CSDH evacuation surgery and therefore increase anesthesia time and operating room time. Further investigation will be necessary to quantify the effect of the increase in operative time on overall patient outcome. However, if CSDH reaccumulation is prevented using the technique described here, return to the operating room would also be prevented and therefore overall operative time, anesthesia time, and hospital length of stay would be greatly reduced. We describe the successful use of dural and outer membrane excision with use of a SEPS drain rather than a traditional rubber catheter drain that either sits on top of the cortical surface or one that is intended to be positioned only a few centimeters into the subdural space through the burr hole. Both of these more traditional drainage methods may potentially cause shearing of bridging veins or cortical vessels thereby increasing the morbidity associated with the operation as well as the rate of re-accumulation of CSDH or even the creation of a new acute SDH.​
